# Car4-positive adipocyte progenitor cells adapt to the aging environment and work as protection against ROS via glutathione metabolism

**DOI:** 10.1038/s41598-025-17231-7

**Published:** 2025-08-29

**Authors:** Masato Horino, Kenji Ikeda, Rei Okazaki, Yujiro Nakano, Ryo Kaneda, Ryoko Ishii, Jun Aoki, Kazunari Hara, Akira Takeuchi, Yoshihiro Niitsu, Kuniyuki Taguchi, Kuniyuki Kano, Masanori Murakami, Kazutaka Tsujimoto, Chikara Komiya, Tetsuya Yamada

**Affiliations:** 1 Department of Molecular Endocrinology and Metabolism, Institute of Science Tokyo, 1-5-45 Yushima, Bunkyo-ku, 113-8510 Tokyo Japan; 2 Department of Endocrinology and Metabolism, Kanazawa University Hospital, 13-1 Takara-machi, Kanazawa, 920-8641 Ishikawa Japan; 3 Department of Diabetes and Endocrinology, Nissan Tamagawa Hospital, 4-8-1 Seta, Setagaya-ku, 158-0095 Tokyo Japan; 4 Department of Health Chemistry, Graduate School of Pharmaceutical Sciences, The University of Tokyo, 7-3-1 Hongo, Bunkyo-ku, 113-0033 Tokyo Japan

**Keywords:** Beige adipocytes, Aging, Adipocyte progenitor cells, Carbonic anhydrase 4, Reactive oxygen species, Glutathione, Endocrine system and metabolic diseases, Cell biology, Endocrinology

## Abstract

The induction of beige adipocytes is significantly reduced in aged mice due to the senescence of adipocyte progenitor cells (APCs). Recent studies have revealed the existence of beige adipocyte subtypes, suggesting that APCs comprise a heterogeneous population. Therefore, in this study, we aimed to elucidate the mechanism through which long-term cold exposure induces the production of beige adipocytes even in aged mice. Single-cell RNA sequencing identified carbonic anhydrase 4 (Car4)-positive APCs. The number of Car4-positive APCs increased with age and cold exposure. Car4 knockdown (KD) mitigated intracellular pH reduction and significantly suppressed beige adipocyte differentiation. Furthermore, Car4 KD cells demonstrated reduced expression of genes in the glutathione pathway and increased susceptibility to reactive oxygen species (ROS), which was alleviated by glutathione supplementation. Our findings suggest that ROS resistance is an adaptation to the cellular aging environment. Our study provides insights into the age-related decline in beige adipocyte induction and identifies Car4 as a potential therapeutic target for enhancing energy expenditure in elderly individuals. This may pave the way for the development of new strategies to combat age-related metabolic diseases and offer hope for improved health and longevity in an aging population.

**Supplementary Information:**

The online version contains supplementary material available at 10.1038/s41598-025-17231-7.

## Introduction

Beige adipocytes, induced by external stimuli, play a crucial role in energy expenditure and systemic metabolism, representing a promising target for treating obesity and type 2 diabetes. Beige adipocytes have also been reported in humans^1,2^, making them viable targets for metabolic therapies. However, the induction of thermogenic adipocytes declines significantly with age, particularly in middle-aged and older individuals. Previous studies have reported that the induction of beige adipocytes is markedly reduced in aged mice owing to the senescence of adipocyte progenitor cells^3^ (APCs), with old mice having impaired cell proliferation and thermogenesis in response to cold exposure^4^. However, these studies were limited to short-term cold exposure; hence, the effects of long-term cold exposure remain unclear. Recent studies have revealed the existence of a subtype of beige adipocytes, suggesting that APCs comprise a heterogeneous population^5^. In the present study, we discovered a new subtype of beige adipocytes induced by long-term cold exposure in aged mice. Our findings indicate that beige adipocytes can be induced by prolonged cold stimulation, even in aged mice. Therefore, this study aimed to elucidate the molecular mechanisms underlying the age-related decline in beige adipocyte induction in order to establish a foundation for therapeutic applications. It is widely known that the amount of reactive oxygen species (ROS) increases in aged cellular environments^6^. Defense mechanisms against ROS are crucial for organisms, with the carbonic anhydrase (Car) family protecting against ROS^7^. Glutathione plays a vital role in the defense against ROS^8^. In this study, we used single-cell analysis to elucidate the diversity of APCs and identify Car4-positive APCs. We found that carbonic anhydrase 4 (Car4) protects APCs against ROS. Thus, Car4-positive APCs are thought to adapt to the cellular environment by increasing their number in response to ROS during aging. Our findings suggest that Car4-positive cells contribute to the induction of beige adipocytes in aged mice. Consequently, our study highlights a potential therapeutic avenue for enhancing energy expenditure in elderly individuals.

## Methods

### Animal experiments

The study received ethical approval from Institute of Science Tokyo ethics committee. All animal experiments were performed in compliance with the ethical regulations of Institute of Science Tokyo (G2019-036C5, A2021-174C5) and the Fundamental Guidelines for Proper Conduct of Animal Experiment and Related Activities in Academic Research Institutions under the jurisdiction of the Ministry of Education, Culture, Sports, Science and Technology of Japan. All methods are reported in accordance with ARRIVE guidelines (https://arriveguidelines.org).

C57BL/6J wild-type (WT) mice were purchased from CLEA Japan, Inc. C57BL/6J-aged mice were obtained from Jackson Laboratory, Japan Inc. All experiments were performed on male mice. All mice were housed under a 12-h light/dark cycle, had free access to regular diet and water, and were caged at an ambient temperature of 23℃. For cold exposure experiments, up to three mice were caged together and exposed to cold temperature (10℃, HC-100, Shin Factory, Japan). To mimic cold conditions, mice were injected intraperitoneally with CL-316,243 (1 mg/kg body weight) for seven consecutive days. Mice were humanely euthanized by cervical dislocation or by intraperitoneal injection (10 ml/kg body weight) of anesthetic mixture containing medetomidine (0.75 mg/kg body weight), midazolam (4 mg/kg body weight), and butorphanol (5 mg/kg body weight).

### Chemicals

The following chemicals were used in this study: Adipose Tissue Dissociation Kit, mouse and rat (130-105-808, Miltenyi Biotec); Adipose Tissue Progenitor Isolation Kit, mouse (130-106-639, Miltenyi Biotec); Dead Cell Removal Kit (130-090-101, Miltenyi Biotec); Hydrogen Peroxide (H_2_O_2_) (081-04215, FUJIFILM Wako Pure Chemical); Click-iT™ Plus EdU Flow Cytometry Assay Kits (C10634, Thermo Fisher Scientific); CyQUANT™ Direct Cell Proliferation Assay (C35011, Thermo Fisher Scientific); 2ʹ,7ʹ-bis-(Carboxyethyl)-5(6ʹ)-carboxyfluorescein Acetoxymethyl Ester (BCECF-AM) (B262, Dojindo); Intracellular pH Calibration Buffer Kit (P35379, Thermo Fisher Scientific); CellROX™ Green Reagent (C10444, Thermo Fisher Scientific); and FluoroBrite™ DMEM (A18967-01, Gibco). The following antibodies were used in this study: Car4 antibody (AF2414, R&D Systems), Dpp4/FITC conjugated antibody (137805, BioLegend), Ucp1 antibody (ab10983, Abcam), α-Tubulin antibody (2144 S, Cell Signaling), Alexa Fluor^®^ 647 (ab150143, Abcam), anti-Rabbit IgG (NA934, GE Healthcare), anti-Goat IgG (ab6885, Abcam), and 4’,6-diamidino-2-phenylindole (DAPI) (17507, AAT Bioquest).

### Metabolic analyses

73-week-old WT mice were exposed to 10℃, while littermate controls were exposed to 23℃, for 4 weeks, and whole-body energy expenditure (VO_2_, VCO_2_) was measured using the ARCO-2000 bioprocess monitoring system (Arco System Inc.).

### Single cell sample preparation

Ten- or 80-week-old WT mice, six each, were randomly assigned to groups of cold (10℃) or an ambient (23℃) temperature for 4 weeks. After collecting inguinal subcutaneous adipose tissue from the mice, the tissue was incubated with collagenase D (Roche, 11088858001) at a concentration of 1.5 U/ml (with 10 mM CaCl_2_) at 37 °C for 45 min. Collagenase D was inactivated by adding 0.5% bovine serum albumin (BSA) / phosphate-buffered saline (PBS), followed by centrifugation at 500×g for 5 min at room temperature. The adipocyte layer in the supernatant was aspirated, and the stromal vascular fractions (SVFs) was suspended in 0.5% BSA/PBS and filtered using a MACS^®^ SmartStrainers 70 μm filter (Miltenyi Biotec, 130-098-462). Preadipocyte and non-preadipocyte fractions were separated using an Adipose Tissue Progenitor Isolation Kit (Miltenyi Biotec, 130-106-639) and LS columns (Miltenyi Biotec, 130-042-401). After washing and resuspending in 0.5% BSA/PBS, the suspension was filtered using a MACS^®^ SmartStrainers 30 μm filter (Miltenyi Biotec, 130-098-458), and dead cells were removed using the Dead Cell Removal Kit (Miltenyi Biotec, 130-090-101) and LS column. Each fraction was adjusted to the concentration specified by 10x Genomics and immediately loaded onto a 10x chromium controller (10x Genomics) according to the manufacturer’s protocol. Quantitative polymerase chain reaction (qPCR) was performed using a Library Quantification Kit (Clontech) to assess library yield, and a 2100 Bioanalyzer DNA 1000 chip (Agilent) was used to determine the library size range and distribution. The library was sequenced in a single lane of a NovaSeq 6000 instrument (Illumina) with 2 × 150 bp paired-end reads using the NovaSeq 6000 S4 Reagent Kit v1.5 (300 cycles).

### Single-cell RNA-seq data analysis

Processing and visualization of single-cell RNA-sequencing (seq) data were performed using the Seurat package (version 4.4.0) in R version 4.3.3 (2024-02-29) (https://satijalab.org/seurat/) on RStudio. The cells were filtered to obtain more than 200 detected genes, less than 6,500 detected genes, and less than 40% of the mitochondrial genome. Expression data were normalized using the normalized data function (scale factor = 1,000,000). The read counts of each cell were regressed as confounding factors within the scale data function. The highly variable genes in each dataset were identified using the FindVariableFeature function. Principal component analysis (PCA) against the identified highly variable genes and projection of PCA onto the entire dataset was performed using the RunPCA function (the number of calculated PCs was 30). Computation of the nearest neighbors was performed using the FindNeighbours function. Cell clustering was performed using the FindClusters function (resolution = 1.5). Dimensionality reduction was performed using a Uniform Manifold Approximation and Projection method (UMAP) by using Seurat. Individual Violin and UMAP plots were generated using the Seurat toolkit’s VlnPlot and FeaturePlot functions, respectively. Differential gene expression among the clusters was analyzed using the FindMarkers function.

Ligand-receptor interaction analysis was performed according to the approach described previously^9^. Briefly, a weighted directed graph was built linking ‘source’ cell types, defined by expression of a ligand, to ‘target’ cell types expressing a corresponding receptor, after reference to a curated map of human ligand-receptor pairs^10^. Source-ligand and receptor-target edges were weighted according to expression fold change in ligands and receptors, respectively. Ligand-receptor edges were weighted according to mouse-specific protein-protein association scores from STRING^11,12^. Significant cell-cell connections were determined by network permutation testing (100,000 permutations, Padj < 0.01).

### Tissue histology

Mice adipose tissues were fixed in 4% paraformaldehyde overnight at 4℃, followed by dehydration in 70% ethanol. The fixed tissues were stored in 70% ethanol until processing at Kyoto Institute of Nutrition and Pathology, Inc.: briefly, tissues were embedded in paraffin and sectioned at a thickness of 4 μm on a silane-coated glass slide. Paraffin-embedded tissues were stained with hematoxylin and eosin (H&E) according to a standard protocol. The images were acquired using an IX73 inverted microscope (Olympus).

### RNA in situ hybridization

RNA in situ hybridization was performed using RNAscope™ 2.5 HD Reagent Kit (Advanced Cell Diagnostics). Fresh paraffin-embedded tissues were prepared according to the manufacturer’s instructions. Briefly, mice adipose tissues were fixed in 10% neutral buffered formalin for 24 h at an ambient temperature of 23 ℃, and the fixed tissues were stored in PBS until processing at Kyoto Institute of Nutrition and Pathology, Inc.: tissues were dehydrated in 70% ethanol, embedded in paraffin, and sectioned at a thickness of 4 μm on a silane-coated glass slide. The paraffin-embedded tissues were deparaffinized twice in xylene and rehydrated. Peroxidase blocking was performed by incubating the tissues for 10 min with RNAscope™ H_2_O_2_ at ambient temperature, after which antigen retrieval was performed by boiling the tissues with RNAscope™ Target Retrieval Buffer for 5 min. After dehydration in 99.5% ethanol, tissues were incubated with RNAscope™ Protease Plus in an oven at 40 ℃ for 15 min. Subsequently, hybridization was performed at 40 ℃ for 2 h using RNAscope™ Probe Mm-*Car4* (Cat No. 468421), followed by several rounds of amplification steps. Finally, chromogenic reactions using RNAscope™ Fast Red and counter-staining with hematoxylin were performed and the slides were mounted with EcoMount (Biocare Medical). Images were captured using an IX73 inverted microscope.

### Isolation of SVF from mouse adipose tissues

Inguinal subcutaneous white adipose tissue (WAT) from WT mice was surgically removed, manually minced into 5 mm^3^ pieces on ice, and placed into gentleMACS C tubes (Miltenyi Biotec) with freshly prepared Dulbecco’s Modified Eagle Medium (DMEM) containing additives included in the adipose tissue dissociation kit. Subsequently, the C Tubes were set into the gentleMACS dissociator with heaters (Miltenyi Biotec) and tissues were digested by running the program 37C_mr_ATDK_1 under 37℃. Digestion was quenched with cold 1 × PBS containing 0.5% BSA and 2 mM ethylenediaminetetraacetic acid (EDTA). The dissociated cells were passed through a 70 μm filter and subjected to centrifugation at 400×g for 5 min under 4℃. The supernatant containing mature adipocytes was decanted, and the pellet containing SVF was resuspended in 1 × PBS buffer containing 0.5% BSA.

### Flow cytometry

SVFs resuspended in fluorescence-activated cell sorting (FACS) buffer were subjected to incubation with the following reagents: non-adipocyte progenitor depletion cocktail for 15 min at 4℃ in darkness, and dead cell removal MicroBeads for 15 min at an ambient condition of 23℃ in darkness. For each procedure, cell suspensions were applied onto LS columns (Miltenyi Biotec) placed in a QuadroMACS™ separator (Miltenyi Biotec) attached to a MACS MultiStand (Miltenyi Biotec), and the flow-through containing unlabeled cells, including viable APCs, was collected. Finally, the cells were incubated with the appropriate antibodies for 30 min at 4℃ in darkness. Flow cytometry analysis and sorting were performed using MoFloXDP (Beckman Coulter).

### Cell proliferation assay

SVFs were isolated from inguinal WAT of aged mice and incubated with 20 µM EdU for 30 min at 37℃. After EdU labeling, cells were stained for Car. EdU-positive cells were subsequently detected using the Click-iT™ EdU Flow Cytometry Assay Kit (Thermo Fisher Scientific) according to the manufacturer’s instructions. Flow cytometry analysis was performed using a FACS Aria Ⅲ (BD Biosciences).

### Cell culture

Immortalized preadipocytes were generated from the SVFs of inguinal subcutaneous WAT of WT mice. Adipogenesis was induced by culturing confluent cells in DMEM containing 10% fetal bovine serum (FBS), 0.5 mM isobutylmethylxanthine, 125 nM indomethacin, 2 µg/ml dexamethasone, 850 nM insulin, 1nM levothyroxine (T_3_), and 0.5 µM rosiglitazone. Two days after adipogenesis induction, the cell medium was replaced with maintenance medium containing 10% FBS, 850 nM insulin, and 1nM T_3_ with/without 0.5 µM rosiglitazone. The maintenance medium was changed every two days. The cells were fully differentiated by day 7 after induction.

### Immunocytostaining

Primary preadipocytes were seeded on a 35 mm glass-bottom dish. Reaching confluency, cells were incubated with Ucp1 Antibody (1:500) and with DAPI at 5 µM at 37℃ for 30 min. Images were captured using an FV3000 inverted microscope (Olympus, Tokyo, Japan).

### Gene knockdown by lentivirus

Lentiviral shRNA constructs targeting mouse *Car4* (CCTTTAGAGGACATGGCTTAT) or a scrambled control (CCTAAGGTTAAGTCGCCCTCG) were purchased from VectorBuilder (*Car4* #VB210515-1011fub, scrambled control #VB010000-0010zkz). For lentivirus production, HEK293T packaging cells were transfected with 6 µg lentiviral plasmids, 3 µg of psPAX2, and 1.5 µg of pMD2. using Lipofectamine™ 3000 according to the manufacturer’s instructions. After 48 h of incubation, the viral supernatants were collected and filtered. Immortalized preadipocytes were incubated with this lentivirus overnight in a medium containing 2 µg/ml polybrene. Hygromycin at 200 µg/ml was used for selecting the stable-expressing cell line. As a green fluorescent protein (GFP) reporter was inserted in the lentiviral shRNA constructs, selection for sorting GFP-positive cells using MoFloXDP was also performed.

### Gene overexpression by lentivirus

The lentiviral mouse *Car4* open reading frame clone expression vector and empty vector control (stuffer) were purchased from VectorBuilder (*Car4* #VB210913-1036agx, empty vector control #VB900120-7563 srw). Lentivirus production and subsequent viral infection of immortalized preadipocytes were performed at Unitech Electronics Co., Ltd. G418 at 5,000 µg/ml was used for selecting the stable-expressing cell line.

### RNA Preparation and quantitative RT-PCR

Total RNA was extracted from mouse subcutaneous WAT, preadipocytes, and mature adipocytes using TRIzol and the RNeasy Mini Kit. cDNA was synthesized using a Veriti Thermal Cycler (Applied Biosystems), according to the manufacturer’s protocol. Quantitative real time (RT)-PCR was performed using the Quant-Studio 6 Flex System (Applied Biosystems). Each sample was run at least in triplicate, and the expression levels of each gene were normalized to *36B4*. Relative mRNA levels were determined by the ∆∆ Ct method. The primer sequences are listed in the Supplementary Information (Suppl. Table S1).

### Western blotting

Car4 over expression (OE) cells and stuffer control cells were lysed in radioimmunoprecipitation assay buffer containing 1% NP-40 and 2 mM protease inhibitors. Total protein lysates (50 µg) were boiled with 6×sample buffer and a partial amount (10 µg) was loaded on a mini-PROTEAN TGX precast gel (Bio-Rad) and subsequently transferred onto an Immuno-Blot^®^ PVDF membrane (Bio-Rad). The blots were blocked in blocking one solution and incubated overnight with Car4 antibody (1:500) or α-Tubulin antibody (1:1000), followed by incubation of secondary antibodies (1:4000) for 1 h at an ambient temperature of 23℃. After reaction with Amersham™ Western blotting detection reagents according to the manufacturer’s instructions, images were captured using a luminescent image analyzer (GE Healthcare).

### Bulk RNA sequencing and bioinformatics

For Car4 knock down (KD) cells and scrambled control cells, total RNA was extracted using TRIzol and RNeasy Mini Kits. For mouse inguinal subcutaneous WAT-derived primary cells, 9- and 61-week-old WT mice were randomly assigned to either an environment of 10℃ or an ambient temperature of 23℃. One week later, the WAT was surgically removed, and SVF isolation was performed as described above. Subsequently, cells were incubated with Car4 antibody (1:100), Dpp4/FITC antibody (1:100), and Alexa Fluor 647 rabbit anti-goat (1:200) at 4℃ for 30 min in the dark, and Car4-positive cells, Dpp4-positive cells, Car4^−^ Dpp4^−^ cells were sorted using MoFloXDP. RNA was isolated from each sorted cell using the TRIzol and miRNeasy Micro Kits. For the bulk-RNA seq cohort, 73-8weeks old wild-type mice were randomly assigned to either a cold environment (10 °C) or an ambient temperature (23 °C). After two weeks of exposure, inguinal subcutaneous white adipose tissue (WAT) was collected for RNA extraction. Sequencing libraries were constructed from the total RNA at DNA Chip Research, Inc. Raw reads for each library were converted to transcripts per million (TPM), and differentially expressed genes (DEGs) were selected for analysis. PCA and hierarchical clustering were performed using the variable genes. Biological pathway analysis was performed using Metascape (https://metascape.org/).

### Cell viability assay

Car4 KD, Car4 OE, and scrambled control cells were seeded at a density of 2,500 cells/well in a black 96-well plate. H_2_O_2_ at different concentrations was added to each group, and cells were incubated under 37℃. After 24 h, numbers of viable cells in each well were measured as relative values to the fluorescent intensity using CyQUANT™ direct cell proliferation assay kit and the multilabel plate reader ARVO X2 2030 (PerkinElmer) according to the manufacturer’s instructions. Cell viability was calculated as follows: (average fluorescent intensity of cells treated with H_2_O_2_ at certain concentration) / (average fluorescent intensity of cells treated with vehicle [H_2_O_2_ 0 µM]). For the time-course assay, H_2_O_2_ at 20 µM was added to each group, and cells were incubated under 37℃ for 2, 4 and 12 h, followed by the assessment of cell viability.

### Intracellular pH assay

Car4 KD or scrambled control cells were seeded on 10-cm dishes. Reaching confluency, cells were treated with or without L-leucyl-L-leucine methyl ester (LLOMe) for 90 min, and then incubated with BCECF-AM at 37℃ for 30 min. After washing with 1×PBS, cells were equally moved to the wells of a black 96-well plate, and pH values were measured relative to the fluorescent intensity using a multilabel plate reader ARVO X2 2030 according to the manufacturer’s instructions. pH calibration was also performed by preparing 2-(4-(2-hydroxyethyl)-1-piperazinyl)-ethanesulfonic acid buffers at pH 6.5-8.0 using an Orion Star A211 Benchtop pH Meter (Thermo Fisher Scientific).

### ROS imaging assay

Car4 KD, Car4 OE, and scrambled control cells were seeded at a density of 100,000 cells/well in 35 mm glass-bottom dishes. Reaching confluency, H_2_O_2_ at 20 µM was added to each group and the cells were incubated for up to 4 h. The production of ROS was visualized with CellROX^®^ green according to the manufacturer’s instructions. Images were captured using an FV3000 inverted microscope.

### Seahorse XFe assay

The oxygen consumption rate (OCR) and extracellular acidification rate (ECAR) of cultured adipocytes were measured using a Seahorse XFe Extracellular Flux Analyzer (Agilent Technologies). Cells were seeded at a density of 30,000 cells/well on the collagen-coated 24-well Cell Culture Microplates (Agilent Technologies) and were maintained in Seahorse XF Assay Medium (Agilent Technologies) supplemented with 1 mM sodium pyruvate, 2 mM GlutaMAX™-Ⅰ, and 25 mM glucose. Cells were pretreated with H_2_O_2_ at 20 µM or vehicle under 37℃ for 1 h, and were subjected to a mitochondrial stress test by adding 5 µM oligomycin.

### Statistics

Statistical analyses were performed using GraphPad Prism Version 10.3.0 (GraphPad Software, Inc.) or IBM SPSS Statistics. All data are presented as means ± standard errors of the mean. The two-tailed ed Student’s *t*-test or analysis of co-variance (ANCOVA) was used for two-group comparisons. The one-way analysis of variance, followed by Tukey’s test, was used for multiple group comparisons. Statistical significance was set at a P-value of less than 0.05.

## Results

### Prolonged cold exposure induces beige adipocytes in aged mice

**Fig. 1:**
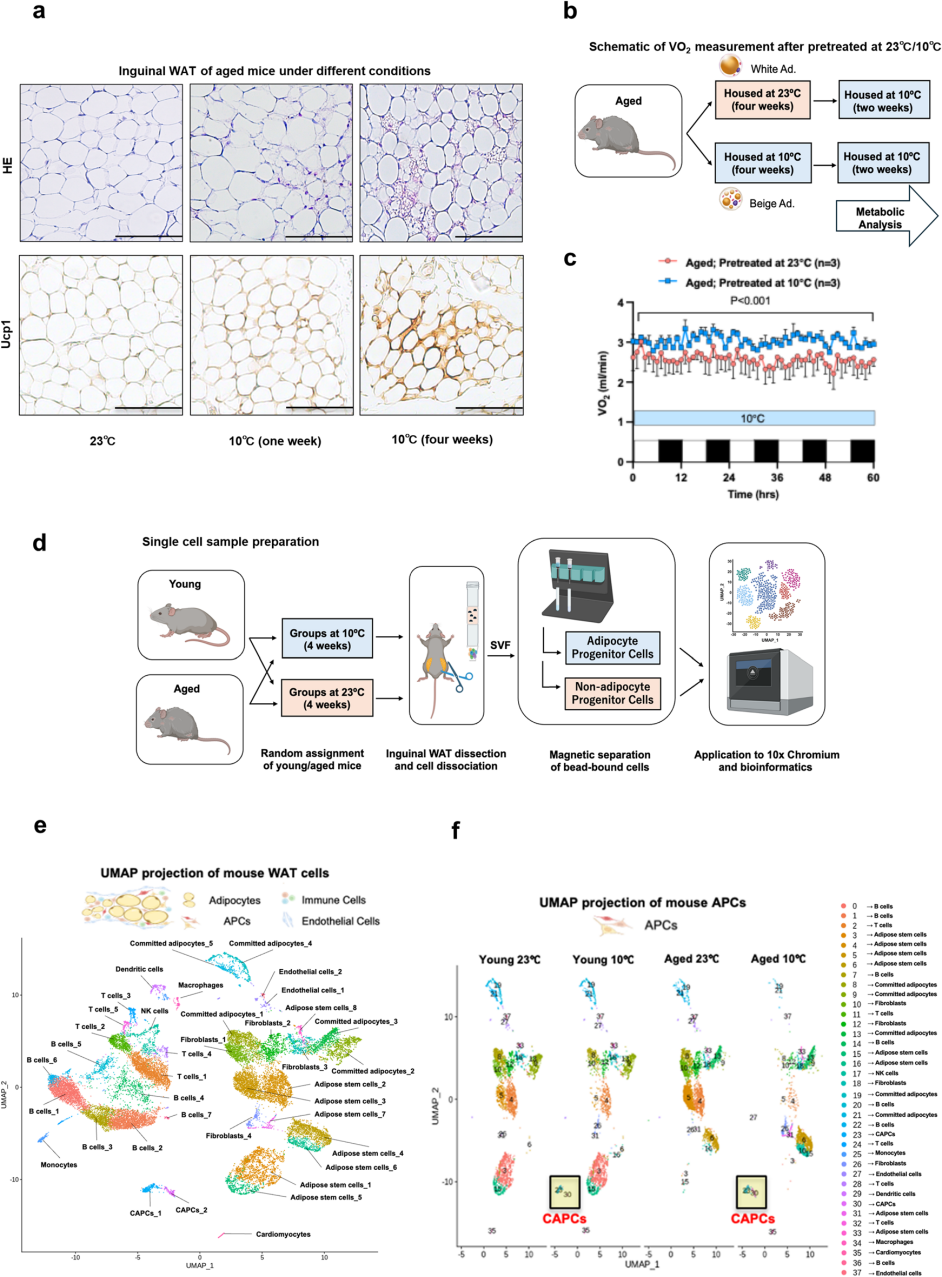
Prolonged cold exposure induces beige adipocytes in aged mice. (a) Representative images of hematoxylin and eosin (H&E) staining (top panels) and Ucp1 immunostaining (bottom panels) in inguinal white adipose tissue (WAT) from aged mice housed at ambient temperature(23℃, left), after 1 week of cold exposure (10℃, middle), or after 4 weeks of cold exposure (10℃, right). Scale bars, 100 μm. (b) Schematic of the metabolic assay with or without cold pretreatment. (c) Whole-body oxygen consumption(VO_2_) of aged mice pretreated at ambient temperature (23℃) or under cold condition (10℃) for 4 weeks. *n* = 3 for both groups. (d) Schematic of sample preparation for single-cell RNA sequencing. (e) Uniform manifold approximation and projection (UMAP) of unsupervised clustering of sequenced mouse WAT cells. (f) UMAP projection of sub-clustering of sequenced adipocyte progenitor cells (APCs), split by age and housing conditions.

Similar to a previous study^3^, aged WT mice did not exhibit beige adipocytes in subcutaneous WAT under an ambient temperature of 23℃ compared with young WT mice. The induction of beige adipocytes by short-term (1 week) cold stimulation was also reduced in aged mice. However, we found that after 4 weeks of prolonged cold stimulation (10℃), induction of beige adipocytes was observed even in aged mice (Fig. 1a). This induction was confirmed by Ucp1 staining which revealed the presence of beige adipocytes (Fig. 1a) and further supported by subcutaneous WAT bulk RNA-seq analysis showing a significant upregulation of *Ucp1* expression in cold-exposed subcutaneous WAT (Suppl. Fig. S1a).

In our study, VO₂ levels were significantly increased in aged mice after 4 weeks of cold exposure compared to age-matched controls maintained at ambient temperature (Fig. 1b–c). Notably, this enhancement in VO₂ remained statistically significant after adjusting for body weight using analysis of covariance (ANCOVA), indicating that the observed metabolic activation is independent of body mass and reflects a genuine physiological response to cold stimulation. Consistent with this interpretation, no significant differences in body weight were observed between the cold-exposed and control groups at the end of the acclimation period (Suppl. Fig. S1b (left)). Interestingly, food intake was markedly elevated in cold-exposed animals (Suppl. Fig. S1b (right)), suggesting that the maintenance of comparable body weight between groups may be due to a compensatory increase in energy intake in response to heightened thermogenic demand.

Together with the upregulation of Ucp1 expression and immunohistochemical evidence of beige adipocyte emergence, these results support the notion that prolonged cold exposure can restore thermogenic competence in aged adipose tissue.

Next, we prepared C57BL/6J WT mice (8–10 weeks old for young, 90–96 weeks old for aged) at 23℃ or 4 weeks with 10℃ cold exposure (*n* = 3 for each group). We isolated SVFs as described previously^5^. Using antibody beads, we separated APCs from other stromal cells and collected the cell populations. Single-cell RNA sequencing was performed using 10x Genomics technology (Fig. 1d). Using the Seurat package (version 4.4.0) in RStudio, we identified 38 distinct cell clusters (Fig. 1e), including APCs, immune cells, and endothelial cells, based on canonical maker gene expression. Each cluster was annotated with a corresponding cell type name (Suppl. Table S2). Single-cell analysis confirmed the presence of specific cold-responsive cell populations (cluster no. 23 and 30) (Fig. 1f).

### Identification of cold-induced apcs

**Fig. 2:**
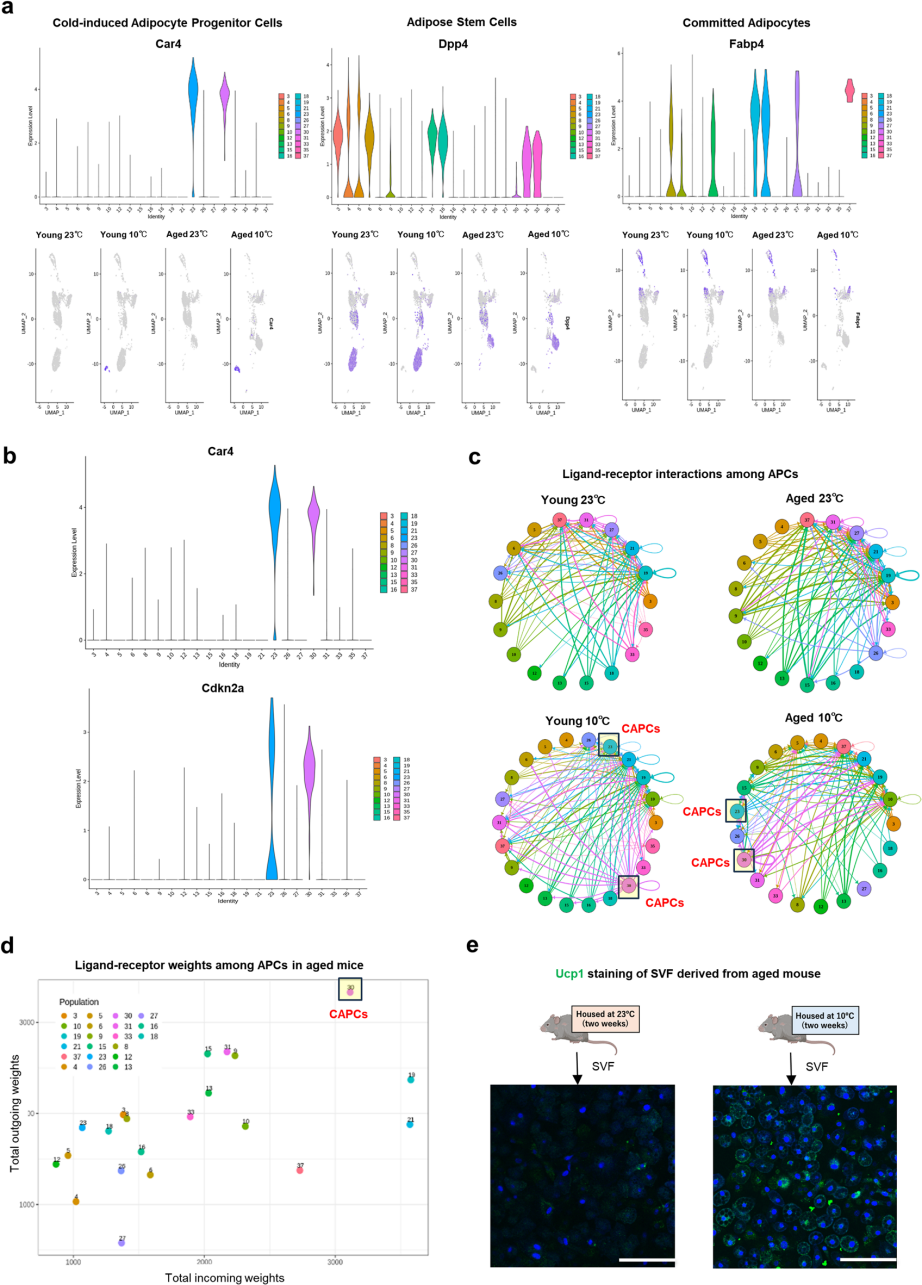
Identification of cold-induced APCs. (a) Violin plots (top) and feature plots (bottom) showing the expression levels and distribution of representative marker genes among APCs. (b) Violin plots Car4 (upper panel) and Cdkn2a (lower panel) gene expression among APC clusters. (c) Intercellular ligand-receptor interaction networks among APCs from young (left) and aged (right) mice under ambient (top) or cold (bottom) conditions. CAPCs are highlighted to indicate enhanced communication within this population. (d) Incoming and outgoing intercellular communication weights among APCs in aged mice under cold conditions. (e) Ucp1 staining of primary SVF cells from aged mice housed at ambient temperature (23℃, left) or after cold exposure (10℃, right). Scale bars, 20 μm.

We identified distinct cold-induced adipocyte progenitor cells (CAPCs) (Fig. 2a). These CAPCs differ from traditional adipose stem cells, which are marked by *Dpp4*, *Pi16*, or *Dmkn*, and from committed adipocytes, which are marked by *Fabp4*,* Plin2*,* or Lpl*^13^. The cells collected using antibody beads in our study were consistent with previously identified APCs^13–15^. Cluster-specific markers for CAPCs were identified (Suppl. Table S3). Notably, both *Car4* and *Cdkn2a* (also known as p16^ink4a^, a well-established cellular aging marker^16^ were selectively expressed in CAPCs, distinguishing this population from other cell clusters (Fig. 2b). Moreover, the single-cell analysis revealed that CAPCs express traditional stem cell markers (e.g., *Cd44*, *Itgb1*) (Suppl. Fig. S2a), suggesting that cold exposure enhances the differentiation capability of SVF cells.

To verify intercellular interactions, we performed ligand-receptor interaction analysis, and the data showed that intercellular interactions among CAPCs were enhanced under cold exposure (Fig. 2c), which may contribute to adipogenic induction, especially in aged mice showing scores of more than 3,000 for both incoming and outgoing weights (Fig. 2d, Suppl. Fig. S2b). These findings underscore the potential of cold exposure as a therapeutic strategy to counteract age-related decline in adipogenic capacity and promote the formation of beige adipocytes. Primary SVF cells cultured after cold exposure for two weeks (10℃) showed increased differentiation into beige adipocytes, as confirmed by Ucp1 staining (Fig. 2e). These results suggest that long-term cold exposure may effectively promote the induction of beige adipocytes, even in aged mice, highlighting its potential as a therapeutic approach.

### Characterization of Car4-positive preadipocytes

**Fig. 3:**
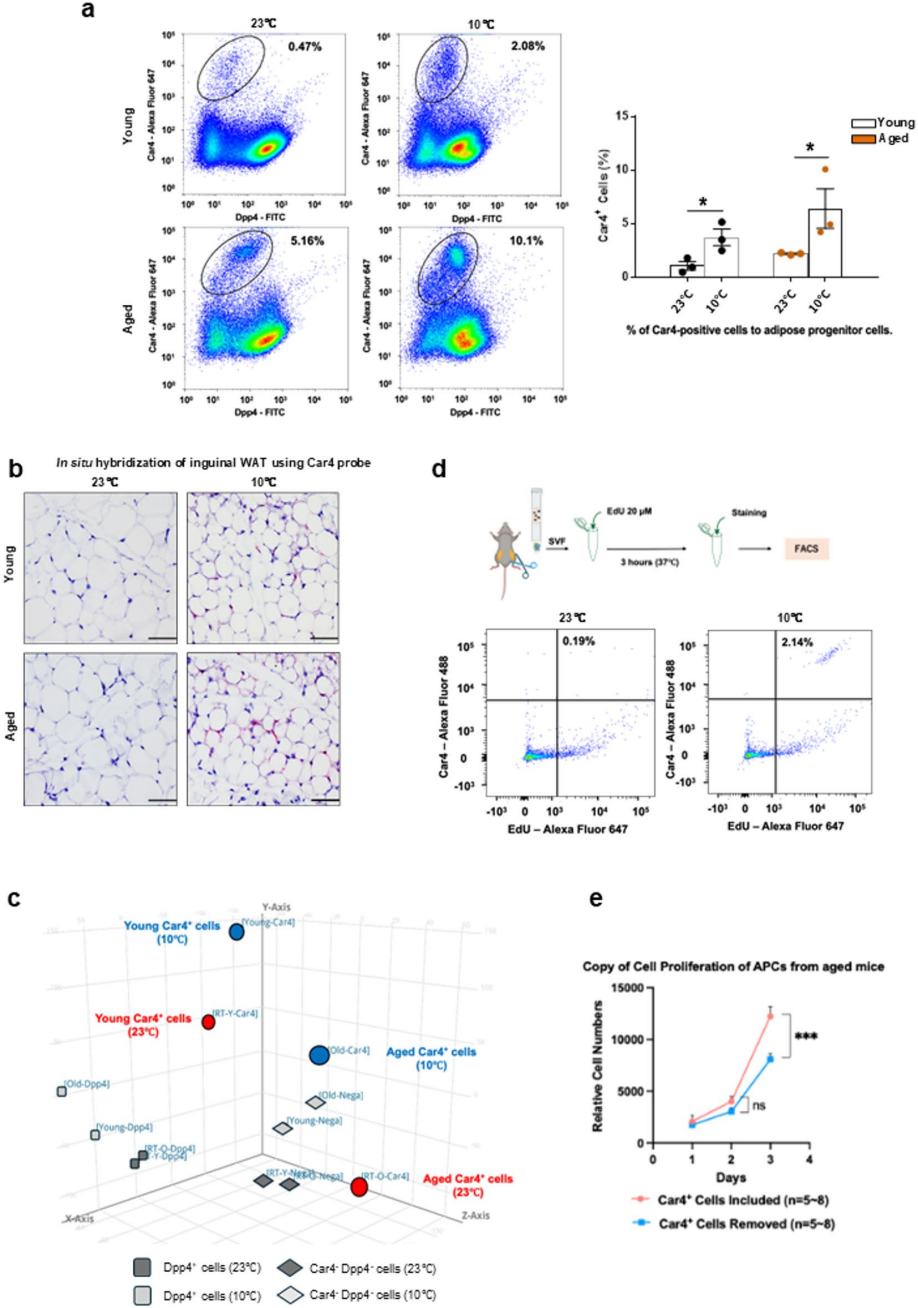
Characterization of Car4-positive preadipocytes. (a) Representative FACS plots (left panes) and quantification (right panel) of Car4-positive cells among APCs in the SVF from inguinal WAT of young (top) or aged (bottom) mice housed under ambient temperature (23℃, left) or cold exposure (10℃, middle). For the quantification, each dot represents an individual biological replicate. Data are presented as mean ± SEM (*n* = 3 per group). *p < 0.05 by one-tailed Student’s t-test. (b) Representative images of RNA in situ hybridization of the inguinal WAT from young (top) or aged (bottom) mice housed at 23℃ (left) or 10℃ (right). (c) Principal component analysis (PCA) score plots of primary Car4-positive cells, Dpp4-positive cells, and Car4^−^Dpp4^−^ cells derived from inguinal WAT of pooled young or aged mice housed at 23℃ or 10℃. (d) Representative FACS plots showing EdU incorporation in Car4-positive preadipocytes from aged mice housed at 23 °C (left) or 10 °C (right). SVF cells were labeled with EdU as described in Methods. Due to the need to pool cells from multiple mice per sample and technical variability in the assay, robust statistical analysis was precluded. This observation, however, is consistent with the Mki67 upregulation shown in Suppl. Fig. S3a. Numbers in plots indicate the percentage of cells in the respective gates. (e) Quantification of APCs proliferation with or without Car4-positive cells. *n* = 5–8 per groups.

To evaluate the cell number of Car4-positive cells in the SVF, we performed FACS using the Car4 antibody, according to a previous study^17^. Flow cytometric analysis of the SVFs revealed a significant increase in the number of Car4-positive cells in aged mice in response to 10℃ cold exposure (Fig. 3a). This observation was confirmed by in situ hybridization of subcutaneous WAT (Fig. 3b). Specifically, the proportion of Car4-positive cells increased from 1.07% at ambient conditions (23℃) to 3.72.% under cold exposure in young mice, while this increase was even more obvious in aged mice, with Car4-positive cells rising from 2.20% at 23℃ to 6.42% under cold exposure (Fig. 3a). These findings suggest that Car4-positive cells play a critical role in adaptive responses to aging and cold stress.

To further elucidate the distinct characteristics of Car4-positive cells, we performed RNA sequencing on Car4-positive cells, Dpp4-positive cells, and Car4 Dpp4 negative cells (Car4^−^Dpp4^−^ cells), followed by PCA. This analysis confirmed that Car4-positive cells possess a unique gene expression profile distinct from that of other cell populations in WAT (Fig. 3c). The unique gene signature of Car4-positive cells underscores their potentially specialized functions within adipose tissues. One of the most notable findings from the gene expression analysis was the increased expression of *Mki67*, a well-known marker of cell proliferation, in Car4-positive cells following cold exposure (Suppl. Fig. S3a). This upregulation was observed not only in young but also in aged mice, indicating that Car4-positive cells retained their proliferative capacity despite aging. Although cell proliferation in adipose tissue is known to decline with age^4^the increased expression of *Mki67* in our study suggests that Car4-positive cells retain the capacity to proliferate in response to cold stress.

To functionally corroborate the upregulation of *Mki67* observed in our RNA-seq analysis, we performed EdU incorporation assays by adding EdU to primary SVF cultures. In SVF derived from room temperature–housed mice, only 0.19% of Car4-positive preadipocytes incorporated EdU. Compared to this, SVF from cold-exposed mice showed a marked increase, with 2.14% of Car4-positive preadipocytes incorporating EdU (Fig. 3d). While technical variability in this primary cell assay precluded robust quantification across replicates, this observation is consistent with the gene expression data and supports the model that cold exposure enhances the proliferative potential of Car4-positive preadipocytes.

To further validate this proliferative capacity using an independent approach, we utilized a β3-adrenergic receptor agonist, CL-316,243 as a mimicked condition of cold exposure in aged mice. After seven day of treatment, APCs were divided into those with or without Car4-positive cells during sorting with MoFloXDP. Subsequently, we compared the proliferation of the two groups and found that APCs with Car4-positive cells were more proliferative than APCS without Car4-positive cells (Fig. 3e). This suggests that Car4 promotes APC proliferation.

Consistent with these functional data, our transcriptomic analysis revealed that signaling pathways related to cell cycle regulation (e.g., cell cycle, cell cycle process, and regulation of cell cycle process) were upregulated in CAPCs under cold conditions compared with ambient conditions in both young and aged mice (Suppl. Fig. S3b).

### Functional analysis of Car4 in apcs

**Fig. 4:**
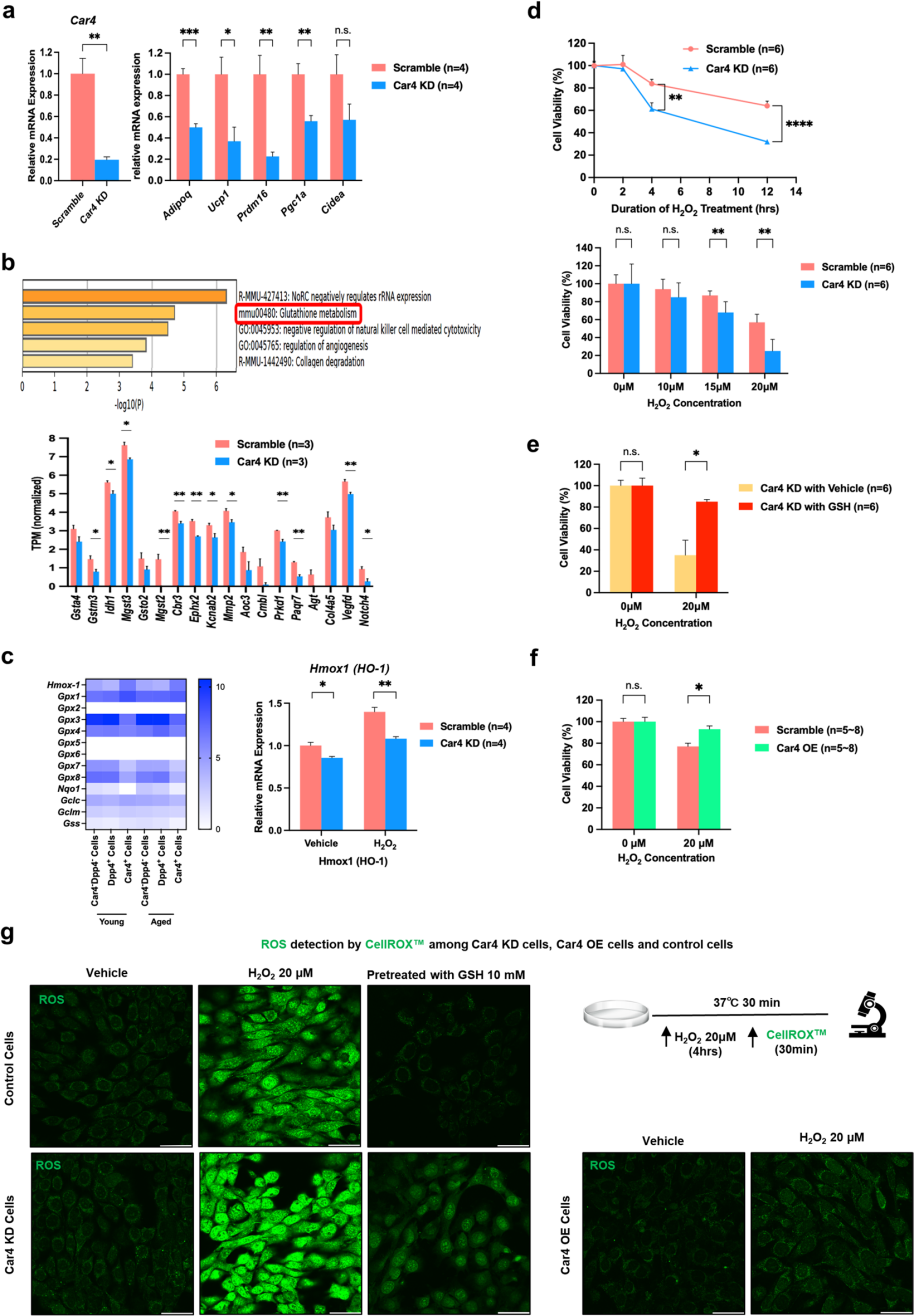
Functional analysis of Car4 in APCs. (a) mRNA expression levels of *Car4* (left) and thermogenic genes (right) in Car4 knockdown (KD) and control (scramble) cells. *n* = 4 per group. (b) Gene ontology (GO) pathways downregulated in Car4 KD cells (top), and quantification of normalized TPM values for genes involved in glutathione metabolism in Car4 KD and control cells (bottom). *n* = 3 per group. (c) mRNA expression of *Hmox-1* in Car4 KD and control cells treated with vehicle (ddH_2_O) or H_2_O_2_ at 20 µM for 4 h, *n* = 4 per group (left). Heatmap showing TPM values of antioxidant gene markers in Car4-positive cells, Dpp4- positive cells, and Car4^−^Dpp4^−^ cells under cold exposure (right). (d) Quantification of cell viability in Car4 KD and control cells treated with H_2_O_2_ (25 µM) for 2, 4, and 12 h (top), or exposed to increasing concentrations H_2_O_2_ for 24 h (bottom). *n* = 6 per group. (e) Cell viability of Car4 KD cells pretreated with or without GSH (10 mM, 30 min) followed by H₂O₂ treatment (20 µM, 24 h). *n* = 6 per group. (f) Cell viability of Car4 overexpressing (OE) and control cells after H₂O₂ treatment (20 µM, 24 h). *n* = 5 (0 µM) and *n* = 8 (20 µM). (g) Representative images of ROS detection in Car4 KD cells (bottom left), Car4 OE cells (bottom right), and control cells (top left) under H_2_O_2_ (20 µM) or vehicle (ddH_2_O) treatment for 2–4 h. GSH (10 mM) was added 30 min before H_2_O_2_ treatment when indicated.

Based on these results, Car4 appears to play a role in beige adipocyte induction. To analyze its function, we generated a Car4 KD APC line. In these cells, the expression of thermogenic genes was significantly decreased during adipogenic differentiation, suppressing the induction of beige adipocytes (Fig. 4a). RNA-seq analysis revealed that Car4 KD cells exhibited downregulated expression of genes related to glutathione metabolism (e.g., *Mgst2*, *Mgst3*, *Gstm3*) (Fig. 4b). Glutathione plays a crucial role in ROS defense by inducing S-glutathionylation (SSG), a redox post-translational modification of proteins that forms disulfide bonds with redox-active cysteines^18^. This modification is essential for protecting cells against oxidative damage. The process of protein SSG is mediated by glutathione and is most effective at an optimal pH^18,19^. Car4 affects the extracellular pH environment, which in turn can lead to the regulation of intracellular pH, because Car4 interacts with transporters^20,21^. Therefore, it is likely that Car4 influences protein SSG and modulates the cellular oxidative stress response.

From the RNA sequencing results on Car4-positive cells, Dpp4-positive cells, and Car4^−^Dpp4^−^ cells, *Gsto1*, which contributes to SSG, showed higher expression in Car4-positive cells than in other cells (Suppl. Fig. S4a). We also found out that *Hmox-1*, an antioxidant gene marker, showed higher expression in Car4-positive cells (Fig. 4c (left)). This was consistent with Car4 KD cells which showed lower expression both under normal (vehicle treatment) and ROS-damaged conditions (H_2_O_2_ treatment) (Fig. 4c (right)). *Hmox-1* can be induced by oxidant stress such as H_2_O_2_, and the function of *Hmox-1* is to protect cells against toxicity by catabolizing free heme^22^. *Gpx1*, a family of glutathione peroxidase, also showed higher expression in Car4-positive cells (Fig. 4c (left)). *Gpx1* can change H_2_O_2_ into H_2_O, a nontoxic form^23^. It is likely that the process of protein SSG by Car4 leads to both *Hmox-1* and *Gpx1* regulation, resulting in cell protection against oxidative damage.

It is hypothesized that Car4 regulates glutathione levels and confers resistance to ROS. To verify this mechanism, we conducted ROS production experiments using H_2_O_2_^24^. In Car4 KD cells, vulnerability to ROS was mitigated by glutathione supplementation (Fig. 4d-e). Furthermore, we generated a Car4 OE APC line (Suppl. Fig. S4b). Car4 OE cells demonstrated increased resistance to ROS induced by H_2_O_2_ (Fig. 4f). These results suggest that Car4 plays a protective role against oxidative stress and highlight its importance in promoting beige adipocyte differentiation.

Next, we analyzed ROS levels inside the cells using an imaging assay^25^. Imaging analysis of intracellular ROS revealed increased ROS level in Car4 KD cells after exposure to H_2_O_2_ (Fig. 4g (left)). Conversely, Car4 OE cells showed significantly reduced ROS production (Fig. 4g (right)). These results indicate that Car4 suppresses ROS production in preadipocytes and inhibits the generation of ROS induced by H_2_O_2_. These results showed consistency between the cell survival data and ROS imaging results.

Overall, our data indicate that Car4 is critical for maintaining the cellular redox balance which in turn supports the differentiation of beige adipocytes and provides resistance against oxidative stress.

### Car4 is involved in the regulation of intracellular pH in preadipocytes

**Fig. 5:**
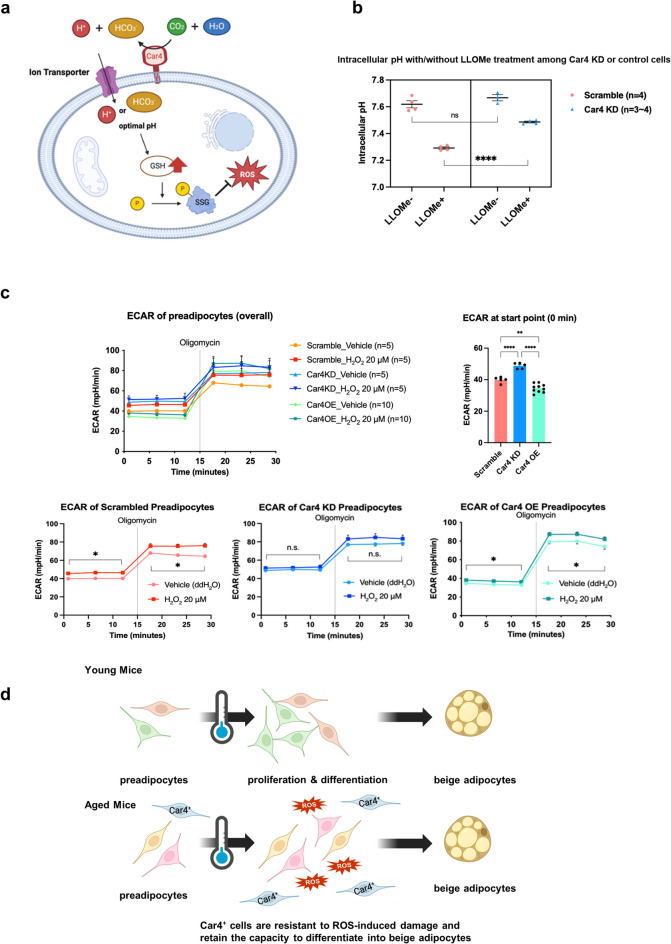
Car4 is involved in the regulation of intracellular pH in preadipocytes. (a) Model of how Car4 plays a protective role against oxidative stress. (b) The intracellular pH of Car4 KD cells and control cells (scramble) with or without L-leucyl-L-leucine methyl ester (LLOMe) treatment (1mM) for 90 min. *n* = 3–4 for each group. (c) The results of extracellular acidification rate (ECAR) (top left) of control cells (bottom left), Car4 KD cells (bottom middle), and Car4 OE cells (bottom right), and the comparison of ECAR of each cells at vehicle condition (top right). *n* = 5–10 for each group. (d) Differential induction of beige adipocytes in young and aged mice. In young mice, preadipocytes exhibit a high proliferative and differentiation into beige adipocytes in response to cold exposure. In contrast, this capacity is diminished in aged mice due to progenitor cell senescence. Notably, Car4⁺ cells, which are induced by prolonged cold exposure in aged mice, are resistant to ROS-induced damage and retain their capacity to differentiate into beige adipocytes.

Based on our experimental results, we developed a hypothetical model in which Car4 plays a role in pH regulation to maintain the balance of redox modifications and protect cells from oxidative stress (Fig. 5a). To further investigate the role of Car4 in cellular pH regulation, we conducted L-leucyl-L-leucine methyl ester (LLOMe) stress experiments. LLOMe damages lysosomes inside cells and induces acidosis of the intracellular pH^26^. In control cells, the intracellular pH decreased because of lysosomal activity. However, in Car4 KD cells, this acidification was less pronounced. Car4 KD cells exhibited a weaker reduction in intracellular acidity after LLOMe treatment than control cells, suggesting that Car4 plays a key role in maintaining intracellular pH acidosis (Fig. 5b).

To further investigate the effect of Car4 on cellular metabolism, we performed a seahorse analysis as previously described^27^. Under normal conditions, Car4 KD cells pretreated with vehicle showed a significantly higher Extracellular acidification rate (ECAR) than control cells. This suggests that Car4 is involved in controlling acid efflux from cells. In contrast, Car4 OE cells exhibited a reduced rate of acid efflux, suggesting that a lower intracellular pH was maintained when Car4 was present, a result that is consistent with the LLOMe experiment results (Fig. 5c). Furthermore, under oxidative stress conditions, both control cell and Car4 OE cells showed increased acid efflux, as indicated by the elevated ECAR following H_2_O_2_ treatment. However, Car4-KD cells did not exhibit an increase in acid efflux (Fig. 5c). These results indicate that Car4 regulates proton movement and acid efflux under both normal and oxidative stress conditions. In addition, we examined oxygen consumption rate (OCR) and found no significant difference between Scramble and Car4 KD cells; however, Car4 OE cells showed a marked increase in OCR. This suggests that Car4 may enhance cellular metabolism through its functional activity (Suppl. Fig. S5a).

Our study showed that the distinct composition of APCs, with Car4-positive cells, which we termed CAPCs, in young and aged mice play a crucial role. These cells may survive and adapt to the aged tissue environment by resisting the increase in ROS levels induced by aging and cold exposure, thereby promoting the induction of beige adipocytes. This cellular adaptation mechanism may be a promising target for future therapeutic strategies. (Fig. 5d)

## Discussion

This study elucidated the functional changes in adipose tissue associated with aging at the single-cell level and identified a novel subtype of APCs expressing Car4. Our findings reveal a new molecular mechanism underlying the age-related decline in beige adipocyte induction and suggest that Car4-positive APCs may play a supportive role in adaptive thermogenesis under cold stimulation in aged individuals.

Car4 is highly expressed in the bone marrow, liver, and gallbladder, whereas low expression is observed in the pancreas, kidney, brain, and adipose tissue^21^. Interestingly, in a recent review, Car4 was listed as a beige adipocyte marker, although the details remain unknown^28^. Previous studies have highlighted the decline in beige adipocyte induction and the associated metabolic impairments with aging. For instance, aging reduces the plasticity of WAT to form beige adipocytes, thereby affecting systemic metabolism^29^. The Car4 expressing subtype of APCs, which we identified in aged mice under cold exposure, suggests the possibility of forming beige adipocytes under aging conditions. This aligns with a previous finding that a subtype of beige adipocytes exists and plays a distinct role in energy metabolism and thermogenesis^27^. In our analysis, Car4 expression was observed not only in the stromal vascular fraction (SVF) but also in mature adipocytes. This raises the possibility that Car4 may have functional roles in differentiated adipocytes as well, although further investigation is required to elucidate its physiological significance in these cells.

Furthermore, our study unveils a critical role for Car4-positive cells as a proliferative hub within the adipose tissue stroma. The observation that the Car4-positive APC fraction exhibits higher proliferative capacity than its Car4-negative counterpart (Fig. 3e) suggests that these cells not only serve as precursors for beige adipocytes but also actively contribute to the expansion of the progenitor pool, especially under conditions like aging and cold stress. This dual potential—acting as both proliferative progenitors and differentiation-competent precursors—positions Car4-positive cells as central players in adipose tissue plasticity and remodeling.

Among the Car family members, Car3 has been reported to exhibit antioxidant effects. Car3 protects cells from H_2_O_2_-induced apoptosis^30,31^. Some studies have suggested that the protein SSG of Car3 itself functions as an oxygen radical scavenger to protect cells^32,33^. Reportedly, SSG is proven to occur only in two members of the Car family, Car3 and Car7^34^. Therefore, it is unclear whether Car4, which we detected, has the same SSG potential as Car3. In contrast, whether Car4 possesses the same modification potential remains unclear. Structurally, Car3 is cytosolic, while Car4 is anchored to the extracellular surface via a glycosylphosphatidylinositol (GPI) anchor^35^. Moreover, while Car4 affects the extracellular and intracellular pH environment via transporters^20,21,36^, a function not known for Car3^37^. These differences suggest that Car4 regulates redox balance via a mechanisms distinct from Car3.

To explore the antioxidant mechanisms associated with Car4-positive cells, our single-cell RNA-seq analysis revealed that, in addition to Hmox1, two key enzymes involved in glutathione metabolism Gsto1 (glutathione S-transferase omega 1) and Gpx1 (glutathione peroxidase 1) were significantly upregulated in Car4-positive cells. Notably, Gsto1 is implicated in the regulation of SSG formation, while Gpx1 catalyzes the reduction of hydrogen peroxide to water. The enrichment of these genes in Car4-positive cells implies that Car4 is linked to regulate redox homeostasis.

The ROS defense system is activated by glutathione which induces protein SSG. This process has been reported to function most effectively at an optimal pH^18,19^. Car4 could affect the regulation of intracellular pH via transporters, which suggests that when intracellular pH reaches an optimal pH, protein SSG can be activated, leading to the protection of cells against oxidative damage^20,21^. Our results showed that the KD of Car4 not only alleviated the reduction in intracellular pH but also significantly suppressed the differentiation of beige adipocytes. This highlights the role of Car4 in regulating intracellular pH and its impact on adipocyte differentiation, suggesting that maintaining a lower intracellular pH is crucial for the differentiation of cells into beige adipocytes. Additionally, the vulnerability to ROS in Car4 KD cells suggests that Car4 is essential for maintaining the cellular redox balance. This was further supported by the finding that glutathione supplementation mitigated the effects of Car4 KD cells.

The identification of Car4 as a key regulator of beige adipocyte differentiation may open new avenues for therapeutic interventions targeting age-related metabolic diseases. Enhancing the function of Car4 or mimicking its activity could improve beige adipocyte function in elderly individuals, thereby enhancing energy expenditure and combating obesity and type 2 diabetes mellitus. Despite these promising findings, our study has a few limitations. First, while we linked Car4 to glutathione metabolism, the precise molecular cascade connecting Car4’s pH-regulating activity to the expression of antioxidant enzymes like Gsto1 and Gpx1 remains to be elucidated. Second, the experiments were conducted primarily in mouse models, and the applicability of the findings to humans remains to be validated. Further research is required to confirm the presence and role of Car4-positive APCs in human adipose tissues. Finally, the long-term effects of Car4 modulation on systemic metabolism and its potential side effects need to be thoroughly investigated before clinical application.

In conclusion, our study offers novel insights into age-related changes in adipose tissue and identifies Car4 as a candidate regulator of beige adipocyte differentiation under cold conditions. While further work is necessary to validate these findings and clarify the underlying mechanisms, Car4-mediated pathways may represent a potential target for future interventions aimed at mitigating metabolic decline in aging individuals.

## Supplementary Information

Below is the link to the electronic supplementary material.

Supplementary Material 1

Supplementary Material 2

Supplementary Material 3

Supplementary Material 4

## Data Availability

The datasets generated and/or analyzed during the current study are available in the DDBJ repository, GEA accession No. E-GEAD-884Access token for reviewer: YFv5DPYbBkw4h4b9uVqo (expired date: 2025-11-13)GEA accession No. E-GEAD-885Access token for reviewer: zCF5Fv6sBaGBxqHkZo0o (expired date: 2025-11-13)GEA accession No. E-GEAD-886Access token for reviewer: O19TKGlmicYeZJ6eb73a (expired date: 2025-11-13).
