# Supplementary material for: Car4-positive adipocyte progenitor cells adapt to the aging environment and work as protection against ROS via glutathione metabolism
Source: Sci Rep. 2025 Aug 29;15:31922. doi: 10.1038/s41598-025-17231-7 (PMC12397281; doi:10.1038/s41598-025-17231-7)
Supplement: Supplementary file 1 — Supplementary Material 1 [file 41598_2025_17231_MOESM1_ESM.pdf]

a

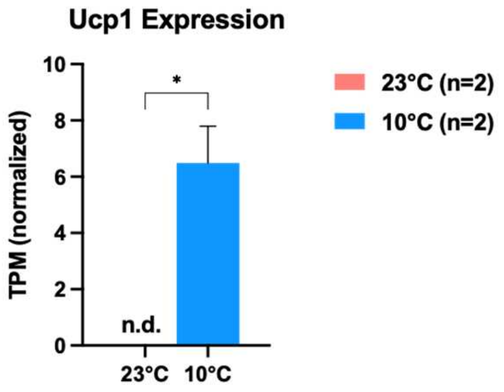

b

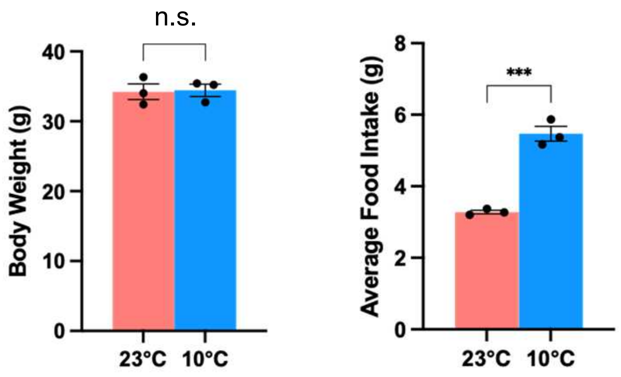

Supplementary Figure S1. **(a)** *Ucp1* expression in inguinal subcutaneous WAT of aged mice maintained at an ambient temperature of 23°C or exposed to 10°C for two weeks. n=2 for both groups. **(b)** Average body weight (left) and food intake (reight) of 73 weeks old mice used for whole-body VO_2 measurements. Mice were acclimated for four weeks at either an ambient temperature of 23°C or a cold condition of 10°C. n=3 for both groups. TPM, transcripts per million. *** P <0.001 by by two-tailed Student's *t*-test. Data are expressed as means ± s.e.m., n.d., not detected. n.s., not significant.

Supplementary Figure S2

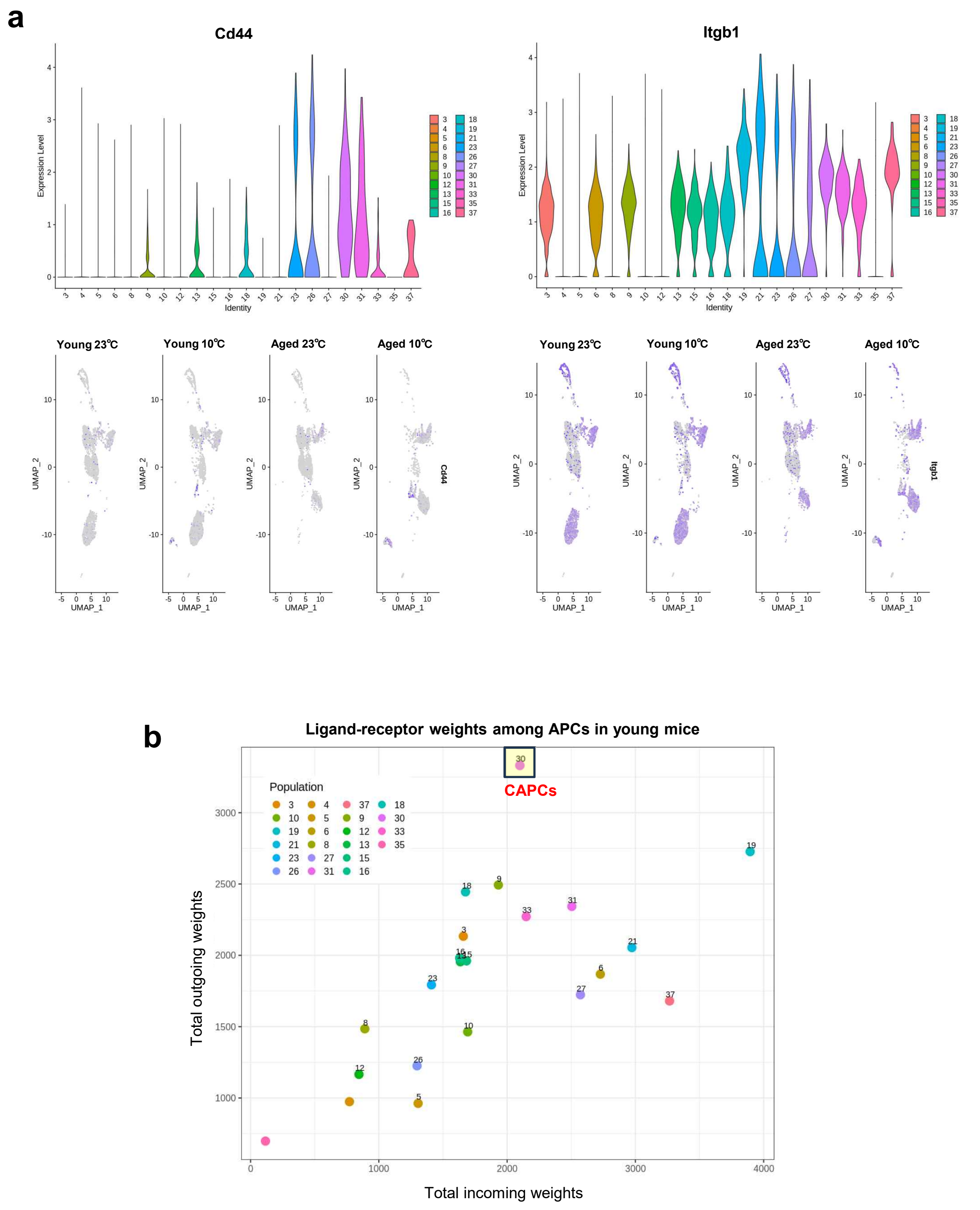

Supplementary Figure S2. (a) Violin plots (top) and feature plots (bottom) showing the expression of stem cell markers in adipocyte progenitor cells. **(b)** The intercellular incoming and outgoing weights among cell clusters of adipocyte progenitor cells from young mice under cold condition.

Supplementary Figure S3

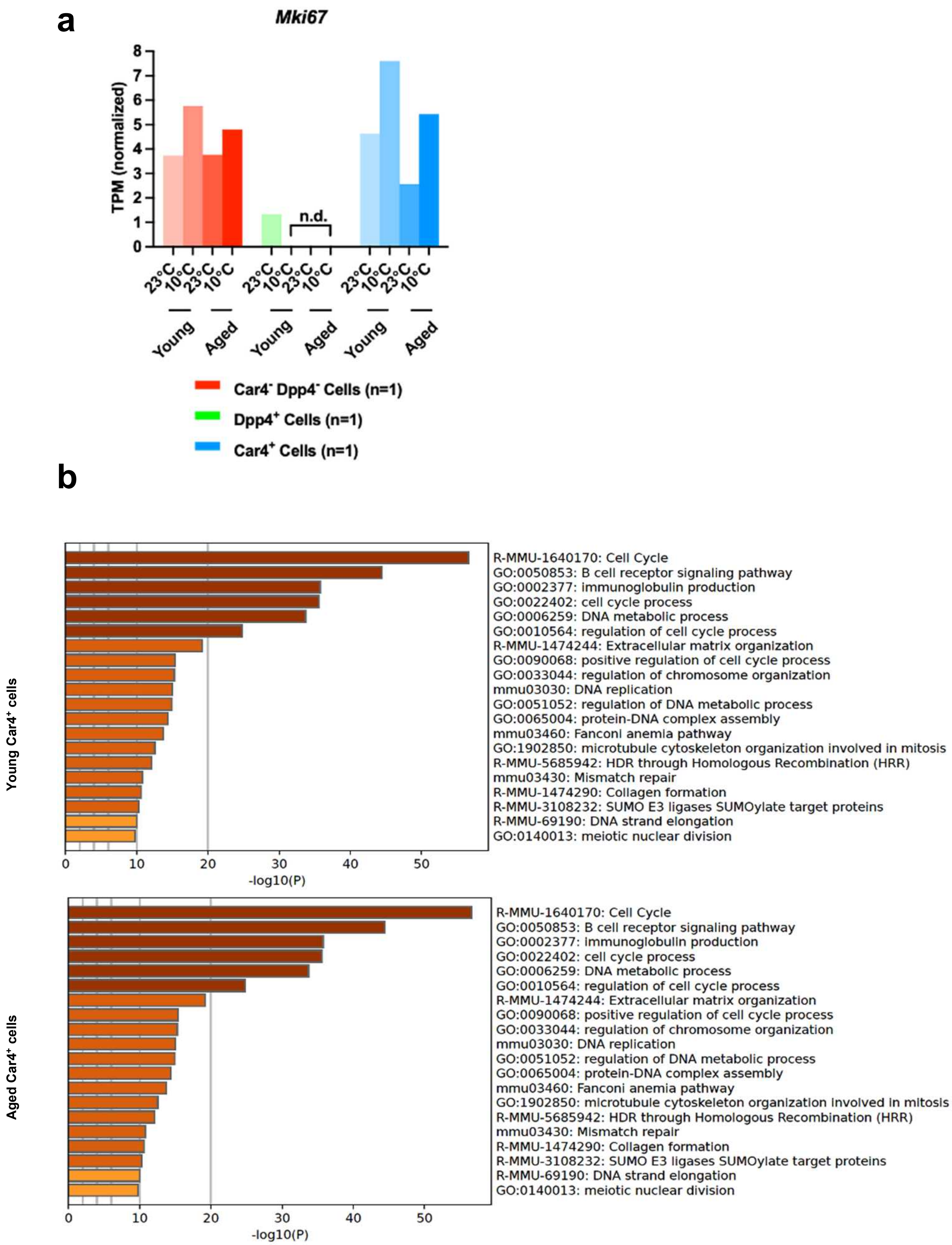

Supplementary Figure S3. (a) Quantification of normalized TPM values of *Mki67* in primary $Car4^+$ cells, $Dpp4^+$ cells, or $Car4^-Dpp4^-$ cells isolated from young or aged mice maintained at either 23°C or 10°C. **(b)** Gene ontology(GO) pathways enriched in primary $Car4^+$ cells from young (top panel) and aged (bottom panel) mice, showing upregulated gene expressions under cold condition(10℃) compared to ambient temperature(23°C). n.d., not detected.

Supplementary Figure S4

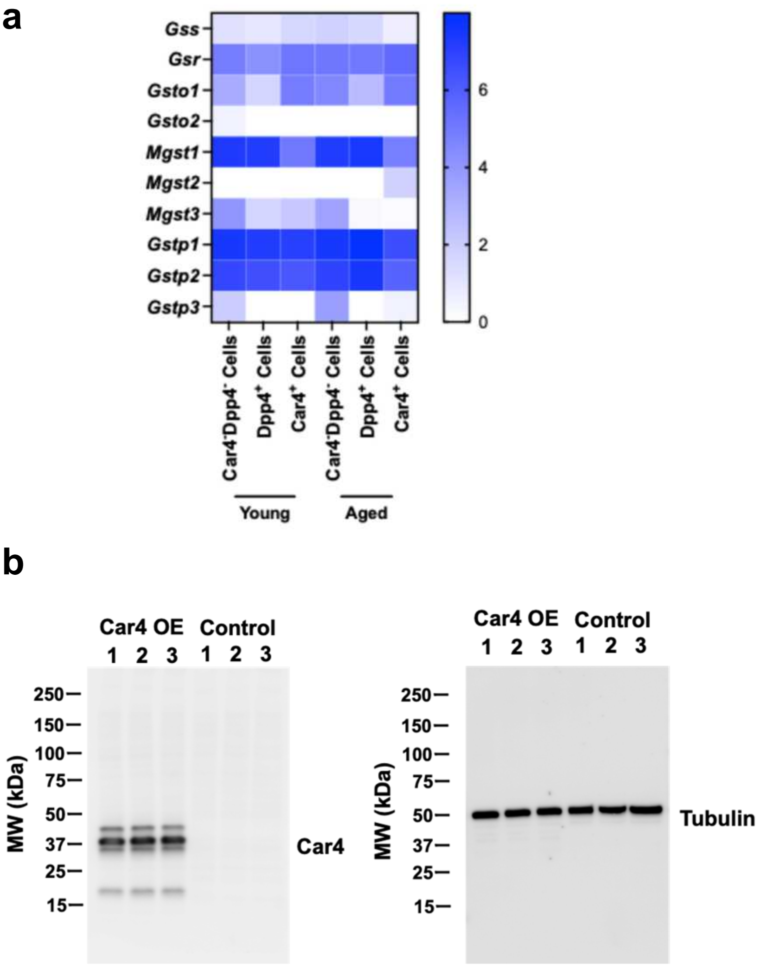

Supplementary Figure S4. **(a)** The heatmap representing TPM values of antioxidant gene markers of Car4-positive cells, Dpp4- positive cells, and $Car4^{-}Dpp4^{-}$ cells under cold condition (10°C).
(b) Western blot detection of Car4 protein (left panel) and tubulin (right panel).
TPM, transcripts per million.

Supplementary Figure S5

a

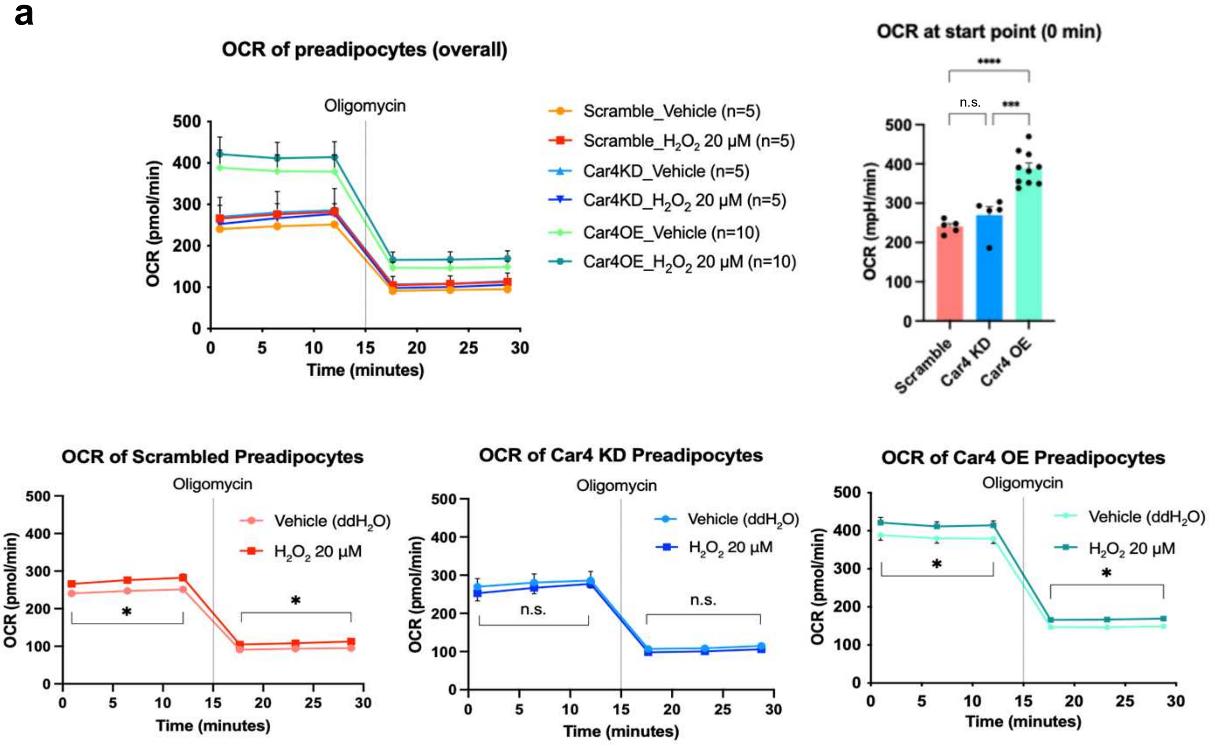

Supplementary Figure S5. **(a)** Oxygen consumption rate (OCR) measurements of control cells (bottom left), Car4 knockdown (KD) cells (bottom middle), and Car4 overexpression (OE) cells (bottom right). The top left panel shows representative OCR traces, and the top right panel summarizes OCR values under vehicle-treated conditions. n = 5–10 per group.

* P <0.05, by by two-tailed Student's *t*-test.

*** P <0.001 by one-way ANOVA followed by Tukey's test. n.s., not significant.

Full-sized Western blot images for Supplementary Figure S6

Car4

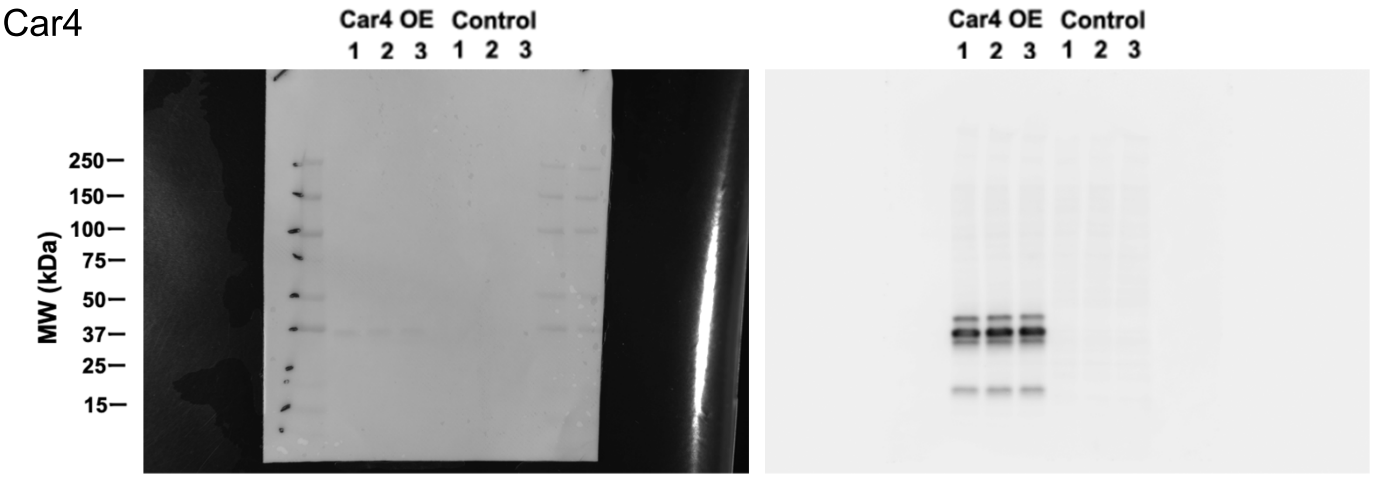

Tubulin

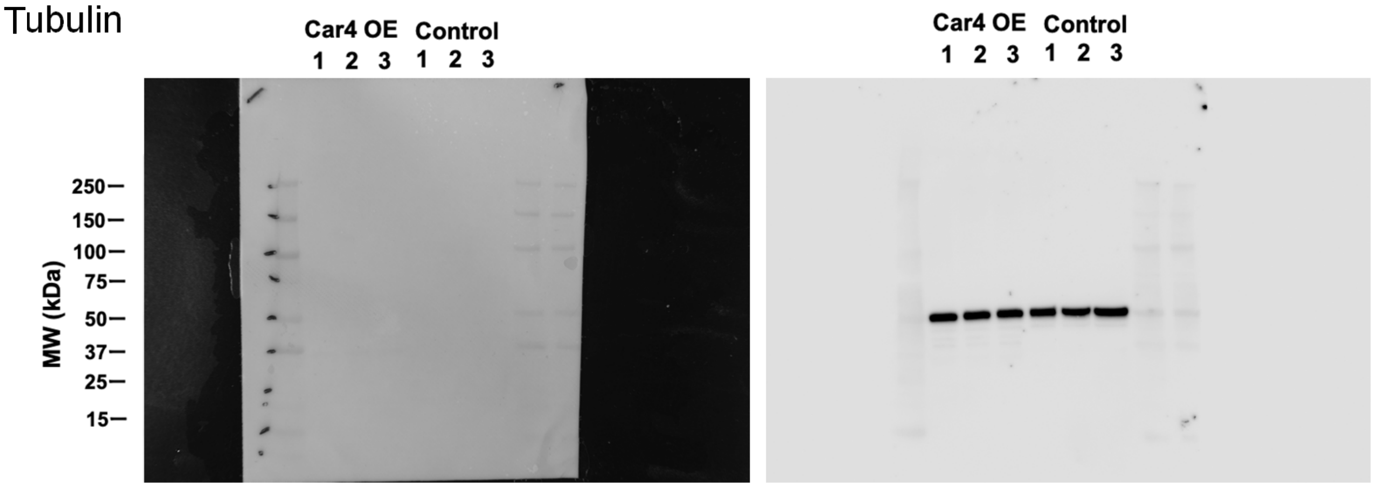

Supplementary Figure S6. Full-sized images of Western blots. For each gene, the two images show the same gel and only the exposure time for scanning differ.
